# N-Terminal proBNP Levels and Tissue Doppler Echocardiography in Acute Rheumatic Carditis

**DOI:** 10.1155/2013/970394

**Published:** 2013-09-11

**Authors:** Alyaa A. Kotby, Ghada S. El-Shahed, Ola A. Elmasry, Iman S. El-Hadidi, Rowaida N. S. El Shafey

**Affiliations:** Faculty of Medicine, Pediatric Department, Ain Shams University, Abbasseya Square, Cairo 11566, Egypt

## Abstract

*Background*. Rheumatic heart disease (RHD) is a leading cause of heart failure in children and young adults worldwide. B-type natriuretic peptide (BNP) is a useful marker of critical pediatric heart disease, and its N-terminal peptide, NT-proBNP, is elevated in congenital and acquired heart disease in children. *Aim*. To measure NT-proBNP levels as a marker of carditis in children with acute rheumatic carditis, as compared to children with quiescent RHD and healthy controls. *Methods*. 16 children with acute rheumatic carditis, 33 children with quiescent RHD, and a cohort of 30 healthy children were studied. Transthoracic echocardiography was performed to assess valve and cardiac function. Tissue Doppler echocardiography was performed for *E/E′* (ratio between mitral inflow *E* wave and lateral mitral annulus *E′* wave) and systolic strain. *Results*. NT-proBNP levels were significantly higher in children with acute rheumatic carditis and dropped with its resolution. Strain and *E/E′* values were comparable among the three groups. *Conclusion*. NT-proBNP is significantly elevated in children with acute rheumatic carditis in the acute stage compared to children with quiescent RHD and healthy subjects, in the presence of comparable echocardiographic indices of LV systolic and diastolic function.

## 1. Introduction

Acute rheumatic fever (ARF) and rheumatic heart disease (RHD) continue to be a major health problem in developing countries, and RHD is the leading cause of heart failure in children and young adults worldwide, resulting in disability and premature death [1]; 80–85% of children younger than 15 years live in areas where rheumatic heart disease is endemic [2].

Late diagnosis is prejudicial since a bout of ARF is a therapeutic emergency. While polyarthritis is the initial and most common major manifestation, carditis is the most serious manifestation of ARF [3] and occurs in around a half of patients [4–9] within 3 weeks of onset of ARF [3]. Although the initial attack can lead to severe valvular disease, ARF might be insidious at onset, and RHD most often results from cumulative valve damage due to recurrent episodes of ARF with a paucity of clinical symptoms [1, 10, 11]. Given that the efficacy and safety of antibiotic prophylaxis are well established and should lead to near complete eradication of advanced RHD when combined with broader changes such as improved living conditions, education, and awareness [12–14], early detection of acute rheumatic carditis will allow appropriate initiation of secondary prophylaxis.

No diagnostic method exists that detects onset of carditis prior to appearance of full-blown clinical or echocardiographic abnormalities [3]. The Jones criteria introduced in 1944 are clinical guidelines for the diagnosis of ARF and carditis, and since then, other criteria have been put forward to increase sensitivity and encourage investigators to standardise patients' characteristics [10, 15, 16]. However, 80 years on, in the absence of any pathognomonic laboratory/imaging test that can establish diagnosis, ARF and acute rheumatic carditis continue to be a clinical diagnosis. Additionally, ARF criteria do not exclude other causes of febrile polyarthritis, which still need to be considered in the differential diagnosis [3], adding further confusion and doubt to the final diagnosis. 

In 2012, the World Heart Federation published updated criteria for echocardiographic diagnosis of RHD. However, these guidelines are not intended for the diagnosis of carditis in the setting of ARF. Whilst echocardiography is valuable in confirming established valve disease, the assessment of its severity, and screening for valve disease in geographic locations with a high prevalence of RHD [17], its role in detection of early carditis in a first episode of ARF or in established RHD is not as well established. 

Brain-type natriuretic peptide (BNP), a member of the natriuretic peptide family, is produced in cardiac myocytes and secreted into the circulation in response to cardiac volume load, causing diuresis, natriuresis, and vasodilatation, as well as inhibition of the renin-aldosterone system and sympathetic activity [18]. BNP is produced as a prohormone that is cleaved in the circulation into active BNP and the N-terminal peptide, NT-proBNP [19]. Both can be assayed, but NT-proBNP has a longer plasma half-life and higher plasma concentrations [20] and therefore can give a good estimate of BNP production. NT-proBNP level was found to be elevated in children with congenital and acquired heart disease, resulting in left and right ventricular volume and/or pressure overload, with the highest levels found in patients with acute LV dysfunction due to acute myocarditis and acute cardiomyopathy, while patients with chronic dilated cardiomyopathy and those with low pressure left to right shunt tended to have the lowest levels [21]. Circulating NT-proBNP levels were also found to be significantly associated with the mitral valve score in patients with chronic rheumatic valve disease and significantly correlated with worsening functional class [22].

Recently, normal values for NT-proBNP concentrations in a large cohort of healthy 8-year-old school children followed over a 4-year period have been published [23]. 

### 1.1. Aim of the Study

The present study was designed to evaluate plasma NT-proBNP as a marker of carditis in patients with acute rheumatic fever, as compared to controlled quiescent RHD and healthy controls. 

## 2. Patients and Methods

This prospective follow-up study was performed over a 2-year period. The study was ethically approved by the Pediatric Department, and consent was obtained from the all children and their parents/carers before enrollment. Seventy-nine children were studied in 3 groups: 16 patients with acute rheumatic carditis who were studied during the acute phase of their illness and after its resolution; 33 patients with known RHD not in activity (quiescent RHD; disease control group); and a cohort of 30 healthy children as a healthy control group.

The diagnosis of acute rheumatic carditis was based on the Jones criteria. ARF was diagnosed when two major or one major and two minor manifestations plus evidence of preceding GAS infection were present. Carditis was defined as the presence of a new murmur, pericardial rub, tachycardia out of proportion to fever, gallop rhythm cardiomegaly, recurrent arrhythmia, and/or congestive heart failure. We excluded patients with infective endocarditis and children who had undergone previous surgical valve repair or replacement of cardiac valves.

All study candidates underwent a thorough medical history, physical examination, routine investigations (FBC, ESR, CRP, and ASOT), 12-lead ECG, and plain chest X-ray. Enzyme immunoassay of plasma NT-proBNP (Biomedica Gruppe; http://www.bmgrp.com) was performed on venous blood samples. 

Transthoracic echocardiography examination (GE medical system—VIVID7 Dimension N-3190—Horten Norway) for all study participants included M-mode measurements, LV end-diastolic and end-systolic volumes, ejection fraction (biplane modified Simpson method), assessment of the degree of mitral regurgitation and aortic regurgitation by colour flow Doppler, and mitral inflow velocities. Pulsed wave tissue Doppler echocardiography was also performed and *E/E*′ calculated. Peak systolic strain and time to peak systolic strain were measured at the left ventricle lateral, septal, anterior, and inferior walls at basal and mid level, and the mean values were calculated. 

### 2.1. Statistical Analysis

Data were collected and analyzed using Student's *t*-test and Mann-Whitney test for comparison of parametric and nonparametric data, respectively, and Pearson and Spearman's tests for detection of correlations in parametric and nonparametric data, respectively. The performance of NT pro-BNP as a predictor of acute rheumatic carditis and the optimal cut-off level were assessed by the ROC curve. A *P* value of less than 0.05 was considered significant.

## 3. Results

The demographic, clinical features, routine laboratory data, medical treatment, and type and severity of valve lesions of the three studied groups are shown in Table 1. In group A, 12 patients had recurrent acute rheumatic carditis, while 7 patients were in their first presentation of acute rheumatic carditis. Eleven patients in the acute rheumatic carditis group (69%) were in NYHA Classes III and IV, compared to none of the quiescent RHD group. All patients with acute rheumatic fever were treated with oral prednisone and salicylates, and 3 patients received packed RBC transfusions for associated anaemia.

### 3.1. Echocardiography

**Figure 1:**
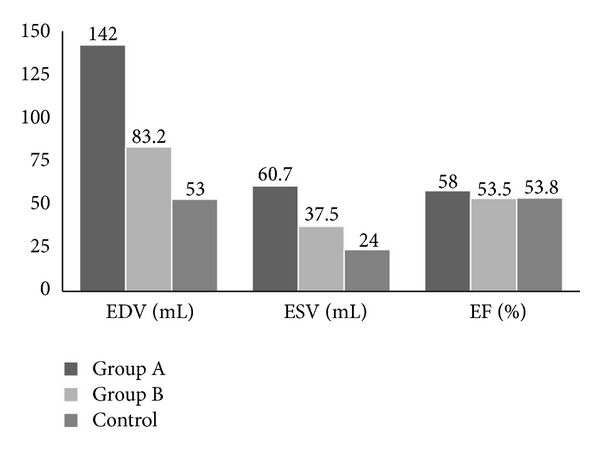
2D echocardiographic data. Patients in acute rheumatic carditis (Group A) had significantly higher EDV than both patients with quiescent RHD (Group B) (*P* < 0.01) and controls (*P* < 0.001) and significantly higher ESV than controls (*P* < 0.001).

**Figure 2:**
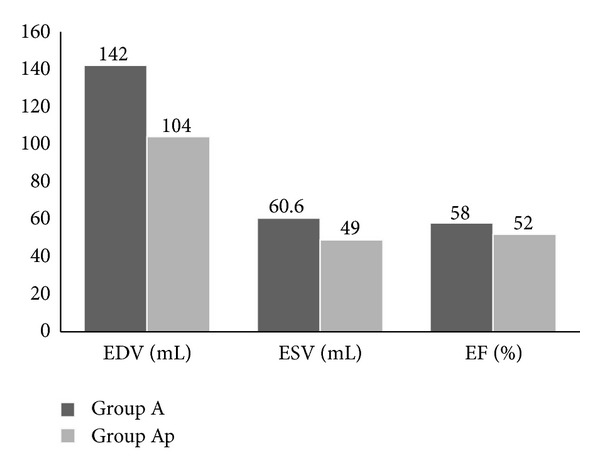
2D echocardiographic data of patients with acute carditis (Group A) during and after resolution (Group Ap) of acute carditis. The drop in values for all 3 parameters was not statistically significant (*P* > 0.05).

Fractional shortening and ejection fraction were not significantly different between groups (*P* > 0.05). Patients in acute rheumatic carditis had significantly higher end-diastolic volumes and end-systolic volumes (Figure 1). Following resolution of the acute carditis, the values of EDV and ESV dropped, but this was not statistically significant (Figure 2).

The *E/E*′ (ratio between the Doppler mitral inflow *E* wave (cm/sec) and the PWTDI lateral mitral annulus *E*′ wave (cm/sec)) was significantly increased in the acute carditis group (10.7 ± 5.3) and quiescent RHD group (7.6 ± 4.2) compared to controls (5.2 ± 1.7) (*P* < 0.01).

**Figure 3:**
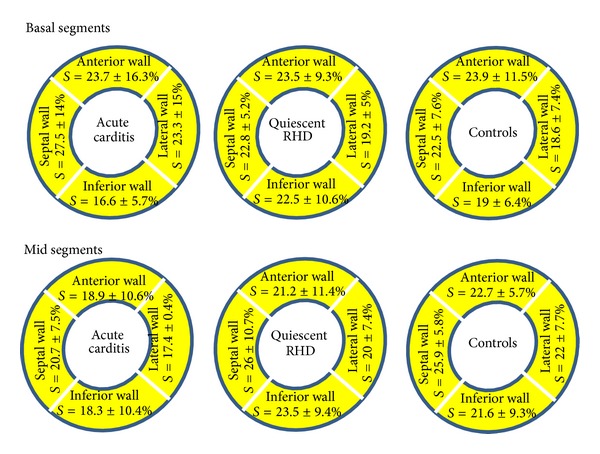
Peak systolic strain in the basal and mid segments of the LV anterior, inferior, lateral, and septal walls in the 3 studied groups.

There was no difference in systolic strain or time to peak systolic strain among the three different groups in the different measured segments or between the mean strain and time to peak strain of all studied 8 LV segments in the 3 groups (*P* > 0.05) (Figure 3).

### 3.2. NT-proBNP Values

**Figure 4:**
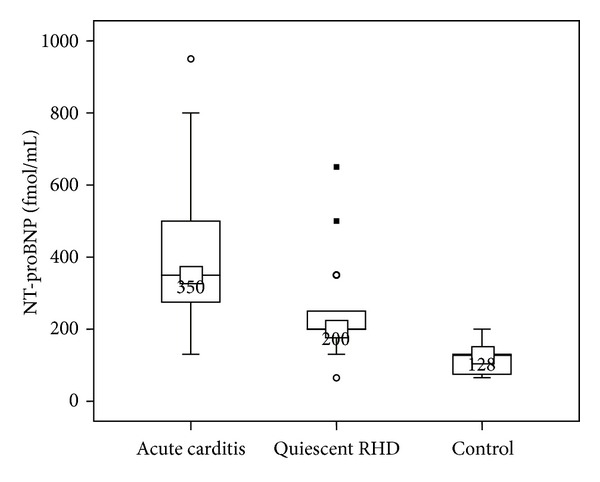
Comparison of plasma NT proBNP in studied groups. Boxes show median and interquartile ranges (IQR) between the 25th and the 75th percentiles. The horizontal line inside the box represents the median, and I bars (lines) represent the highest and lowest values (range of concentrations). The small closed squares represent extreme values (more than 3 IQR), and small open circles represent the outlier values (between 1.5 and 3 IQR).

Patients in acute rheumatic carditis had significantly higher NT-proBNP values than patients with quiescent RHD and controls (*P* < 0.001). Similarly, patients with quiescent RHD had significantly higher NT-proBNP values than controls (*P* < 0.001) (Table 1; Figure 4). There was no significant difference in NT-proBNP levels between patients with acute rheumatic carditis presenting in their first attack and those with recurrent rheumatic carditis (*P* > 0.05).

We found a significant positive correlation between NT proBNP levels and *E/E*′ ratio (*r* = 0.308; *P* < 0.05), EDV (*r* = 0.55; *P* < 0.001) and ESV (*r* = 0.57; *P* < 0.001).

**Figure 5:**
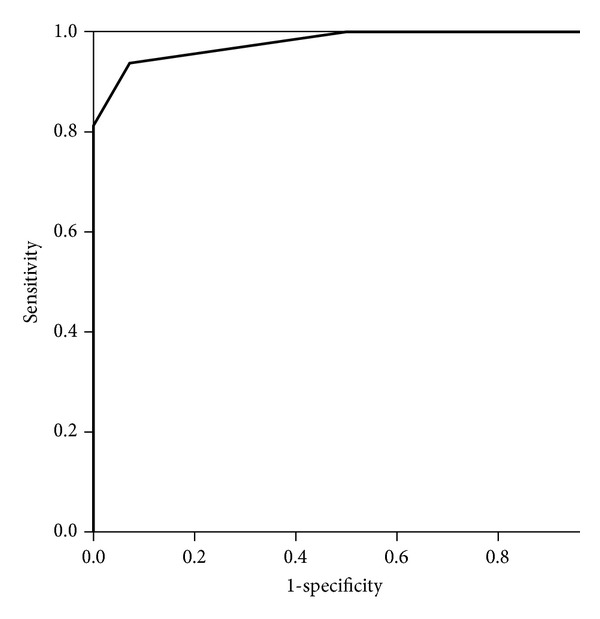
Receiver-operating characteristic (ROC) curve for plasma NT-proBNP in the diagnosis of acute rheumatic carditis. The area under the curve was 0.978. A cut of value of 265 fmol/mL has a sensitivity of 93% and specificity of 92.9% in detecting acute rheumatic carditis.

Following resolution of the acute rheumatic carditis, mean plasma NT-proBNP levels dropped (174.6 ± 65 fmol/mL; *P* < 0.001), but was still significantly higher than the control group (*P* < 0.001). An NT-proBNP plasma level of 265 fmol/mL had a sensitivity of 93% and a specificity of 92.9% in detecting acute carditis in patients with acute rheumatic fever using the ROC curve. The area under the curve was 0.978 (Figure 5).

## 4. Discussion

NT-proBNP was significantly increased in children with acute rheumatic carditis compared both to children with quiescent RHD and healthy controls. Many of these patients were in heart failure (NYHA class II–IV) secondary to rheumatic carditis and worsening valve lesions, with resultant volume overload as evidenced by significantly increased EDV and ESV. NT-proBNP levels dropped significantly after resolution of the acute carditis and improvement of heart failure and volume overload secondary to treatment. Natriuretic peptide levels are known to be reduced by treatment [24].

Similar to other studies, the various conventional echocardiographic parameters measured in this study did not help to differentiate between acute rheumatic carditis and established chronic rheumatic heart disease without carditis. Clinical studies using spectral tissue Doppler for recording mitral annular velocities have produced convincing evidence of a positive, linear relation of *E/E*′ with invasively determined mean LV diastolic pressure regardless of LV ejection fraction, rhythm, and heart rate [25–27]. In our patients, there was no evidence of diastolic dysfunction as evidenced between the lack of any significant difference between patient groups and controls in measured *E/E*′.

LV strain has been hailed as a very sensitive and volume-independent modality for assessment of myocardial function. Strain imaging has been used to assess myocardial function in a wide range of cardiac conditions. It is useful in detecting early left ventricular (LV) dysfunction in the setting of systemic diseases with cardiac involvement, in differentiating transmural from nontransmural infarction and in identifying LV contractile reserve in regurgitant valve lesions [28]. There was no significant difference in measured LV strain amongst our patient groups and in comparison to the control group. The echocardiographic data in our patients suggests that rheumatic myocarditis, reflected as impaired diastolic and/or systolic function, is not apparent in this patient group and that the elevated NT-proBNP was due to uncontrolled heart failure as a consequence of the entire “pancarditis” process, of which rheumatic valve affection was probably the major contributor. The improvement of said process was associated with a decline in NT-proBNP.

Measurement of natriuretic peptides, used in conjunction with other clinical information, has been proven useful as a diagnostic marker to aid in the recognition of pediatric critical heart disease in the acute care setting [29]. Rheumatic carditis occurs a few weeks after the initial infection in about 50% of patients with acute rheumatic fever and presents as valvulitis, sometimes combined with pericarditis or myocarditis [6, 30]. Given the results of the current study, NT-proBNP assay might be a useful adjunct to the diagnosis of rheumatic carditis in patients presenting with noncardiac signs of rheumatic fever. Baseline levels at initial presentation followed by weekly serial measurements may help detect the early onset of carditis in patients before the emergence of the more florid clinical signs of carditis. 

We found that a cut-off level of 265 fmol/mL had a reasonable high sensitivity and specificity for detecting acute carditis in the setting of RF and RHD; however, there was wide overlap in NT-proBNP values between the acute carditis group and the quiescent RHD group. Whilst elevated NT proBNP might also be a consequence of worsening mechanical heart failure or complicating infective endocarditis in a patient with established rheumatic valvular disease, it could potentially be highly suggestive of acute carditis in patients without history of rheumatic heart disease but presenting with one of the major noncardiac criteria of rheumatic fever. 

Given the paucity of investigations that detect early carditis in this patient population, the absence of any specific echocardiographic findings prior to onset of valve regurgitation, and the potential value of treatment in early rheumatic carditis [31], this can prove to be a valuable objective investigation to be added to the Jones criteria.

## Figures and Tables

**Table 1:** Data of the studied groups.

Variable	Acute rheumatic carditis *n* = 16	Quiescent RHD *n* = 33	Control group *n* = 30

Age (yrs)			
Mean ± SD (range)	12.56 ± 1.9 (8.0–15)	12.3 ± 2.73 (6.0–16)	11.9 ± 2.9 (6.0–16)
Males			
*n*	10	18	17
Weight (Kg)			
Mean ± SD (range)	41.5 ± 16 (25–85)	39.9 ± 15.8 (20–70)	34.2 ± 12.7 (17–55)
Height (cm)			
Mean ± SD (range)	145.7 ± 12.2 (125–163)	142 ± 15.4 (109–171)	138.1 ± 18.8 (107–19)
NYHA			
I	4	24	0
II	1	9	0
III	3	0	0
IV	8	0	0
Arthritis	12	0	0
Chorea	2	0	0
Medications			
Furosemide	12	5	0
Spironolactone	4	0	0
Digoxin	10	4	0
Captopril	10	15	0
Haloperidol	2	0	0
Prednisolone	16	0	0
Acetyl salicylic acid	16	0	0
Valve lesions			
Isolated MR	4	18	0
Isolated AR	1	0	0
MR and AR	11	15	0
Severe MS	0	2	0
			
Severity of valve lesions			
MR			
Mild	3	23	0
Moderate	7	9	0
Severe	5	1	0
AR			
Mild	7	10	0
Moderate	3	5	0
Severe	2		0
Pericardial effusion	11	1	0
Mean LV strain (%)			
Mean ± SD (range)	20.9 ± 8.6 (12–49)	22 ± 3 (14–28)	21 ± 3.4 (17–27.7)
Mean LV time to peak strain (sec)			
Mean ± SD (range)	0.3 ± 0.05 (0.14–0.36)	0.32 ± 0.02 (0.31–0.35)	0.32 ± 0.01 (0.3–0.37)
NT-proBNP (fmol/mL)			
Mean ± SD (range)	420 ± 223 (130–950)	247 ± 106.4 (65–650)	116 ± 37 (65–200)

Abbreviations: BSA: body surface area; NT-proBNP: N-terminal pro-brain-type natriuretic peptide; NYHA: New York Heart Association functional class; RBCs: red blood cells; RF: rheumatic fever; SD: standard deviation; WBCs: white blood cells.

## References

[1] Carapetis JR, Steer AC, Mulholland EK, Weber M (2005). The global burden of group A streptococcal diseases. *The Lancet Infectious Diseases*.

[2] Population Reference Bureau world population data sheet. http://www.prb.org/Publications/Datasheets/2008/2008wpds.aspx.

[3] Weil-Olivier C Rheumatic fever. Orphanet encyclopedia. http://www.orpha.net/data/patho/GB/uk-RF.pdf.

[4] Meira ZMA, Goulart EMA, Colosimo EA, Mota CCC (2005). Long term follow up of rheumatic fever and predictors of severe rheumatic valvar disease in Brazilian children and adolescents. *Heart*.

[5] Caldas ÁM, Terreri MTRA, Moises VA (2008). What is the true frequency of carditis in acute rheumatic fever? A prospective clinical and doppler blind study of 56 children with up to 60 months of follow-up evaluation. *Pediatric Cardiology*.

[6] Sanyal SK, Thapar MK, Ahmed SH (1974). The initial attack of acute rheumatic fever during childhood in North India. A prospective study of the clinical profile. *Circulation*.

[7] Vardi P, Markiewicz W, Weiss Y (1983). Clinical-echocardiographic correlations in acute rheumatic fever. *Pediatrics*.

[8] Vasan RS, Shrivastava S, Vijayakumar M, Narang R, Lister BC, Narula J (1996). Echocardiographic evaluation of patients with acute rheumatic fever and rheumatic carditis. *Circulation*.

[9] Figueroa FE, Valdés P, Carrión F (2001). Prospective comparison of clinical and echocardiographic diagnosis of rheumatic carditis: long term follow up of patients with subclinical disease. *Heart*.

[10] Carapetis JR, McDonald M, Wilson NJ (2005). Acute rheumatic fever. *The Lancet*.

[11] Sliwa K, Carrington M, Mayosi BM, Zigiriadis E, Mvungi R, Stewart S (2010). Incidence and characteristics of newly diagnosed rheumatic heart disease in Urban African adults: insights from the Heart of Soweto Study. *European Heart Journal*.

[12] Markowitz M, Kaplan E, Cuttica R (1991). Allergic reactions to long-term benzathine penicillin prophylaxis for rheumatic fever. *The Lancet*.

[13] Nordet P, Lopez R, Dueñas A, Sarmiento L (2008). Prevention and control of rheumatic fever and rheumatic heart disease: the Cuban experience (1986–1996–2002). *Cardiovascular Journal of Africa*.

[14] Arguedas A, Mohs E (1992). Prevention of rheumatic fever in Costa Rica. *Journal of Pediatrics*.

[15] National Heart Foundation of Australia and the Cardiac Society of Australia and New Zealand Diagnosis and management of acute rheumatic fever and rheumatic heart disease in Australia: an evidence-based review. http://www.heartfoundation.org.au/SiteCollectionDocuments/Diagnosis-Management-Acute-Rheumatic-Fever.pdf.

[16] Carapetis JR, Paar J, Cherian T Standardization of epidemiologic protocols for surveillance of post-streptococcal sequelae: acute rheumatic fever, rheumatic heart disease and acute post-streptococcal glomerulonephritis. http://www.niaid.nih.gov/topics/strepThroat/Documents/groupasequelae.pdf.

[17] Reméanyi B, Wilson N, Steer A (2012). World Heart Federation criteria for echocardiographic diagnosis of rheumatic heart disease-an evidence-based guideline. *Nature Reviews Cardiology*.

[18] Espiner EA, Richards AM, Yandle TG, Nicholls MG (1995). Natriuretic hormones. *Endocrinology and Metabolism Clinics of North America*.

[19] Luchner A, Stevens TL, Borgeson DD (1998). Differential atrial and ventricular expression of myocardial BNP during evolution of heart failure. *American Journal of Physiology*.

[20] Rodeheffer RJ (2004). Measuring plasma B-type natriuretic peptide in heart failure: good to go in 2004?. *Journal of the American College of Cardiology*.

[21] Nir A, Bar-Oz B, Perles Z, Brooks R, Korach A, Rein AJJT (2004). N-terminal pro-B-type natriuretic peptide: reference plasma levels from birth to adolescence. Elevated levels at birth and in infants and children with heart diseases. *Acta Paediatrica, International Journal of Paediatrics*.

[22] Davutoglu V, Celik A, Aksoy M, Sezen Y, Soydinc S, Gunay N (2005). Plasma NT-proBNP is a potential marker of disease severity and correlates with symptoms in patients with chronic rheumatic valve disease. *European Journal of Heart Failure*.

[23] Koerbin G, Abhayaratna WP, Potter JM, Apostoloska S, Telford RD, Hickman PE (2012). NTproBNP concentrations in healthy children. *Clinical Biochemistry*.

[24] Maisel A, Mueller C, Adams K (2008). State of the art: using natriuretic peptide levels in clinical practice. *European Journal of Heart Failure*.

[25] Ommen SR, Nishimura RA, Appleton CP (2000). Clinical utility of Doppler echocardiography and tissue Doppler imaging in the estimation of left ventricular filling pressures: a comparative simultaneous Doppler-catheterization study. *Circulation*.

[26] Sundereswaran L, Nagueh SF, Vardan S (1998). Estimation of left and right ventricular filling pressures after heart transplantation by tissue doppler imaging. *American Journal of Cardiology*.

[27] Bruch C, Grude M, Müller J, Breithardt G, Wichter T (2005). Usefulness of tissue Doppler imaging for estimation of left ventricular filling pressures in patients with systolic and diastolic heart failure. *American Journal of Cardiology*.

[28] Leung DY, Ng ACT (2010). Emerging clinical role of strain imaging in echocardiography. *Heart Lung and Circulation*.

[29] Maher KO, Reed H, Cuadrado A (2008). B-type natriuretic peptide in the emergency diagnosis of critical heart disease in children. *Pediatrics*.

[30] Kamblock J, Payot L, Iung B (2003). Does rheumatic myocarditis really exists? Systematic study with echocardiography and cardiac troponin I blood levels. *European Heart Journal*.

[31] Herdy GV, Gomes RS, Silva AE, Silva LS, Lopes VG (2012). Follow-up of rheumatic carditis treated with steroids. *Cardiology in the Young*.

